# A randomised controlled trial of the clinical and cost-effectiveness of ultrasound-guided intra-articular corticosteroid and local anaesthetic injections: the hip injection trial (HIT) protocol

**DOI:** 10.1186/s12891-018-2153-0

**Published:** 2018-07-18

**Authors:** Zoe Paskins, Gemma Hughes, Helen Myers, Emily Hughes, Susie Hennings, Andrea Cherrington, Amy Evans, Melanie Holden, Kay Stevenson, Ajit Menon, Kieran Bromley, Philip Roberts, Alison Hall, George Peat, Clare Jinks, Raymond Oppong, Martyn Lewis, Nadine E. Foster, Christian Mallen, Edward Roddy

**Affiliations:** 1 Arthritis Research UK Primary Care Centre, Research Institute for Primary Care & Health Sciences, Keele University, Staffordshire, ST5 5BG UK; 2 Haywood Academic Rheumatology Centre, Midlands Partnership NHS Foundation Trust, Stoke-on-Trent, UK; 3 Keele Clinical Trials Unit, Keele University, Newcastle-under-Lyme, UK; 4 University Hospitals North Midlands, Stoke-on-Trent, UK; 5 Health Economics Unit, University of Birmingham, Birmingham, UK

## Abstract

**Background:**

Evidence on the effectiveness of intra-articular corticosteroid injection for hip osteoarthritis is limited and conflicting. The primary objective of the Hip Injection Trial (HIT) is to compare pain intensity over 6 months, in people with hip OA between those receiving an ultrasound-guided intra-articular hip injection of corticosteroid with 1% lidocaine hydrochloride plus best current treatment with those receiving best current treatment alone. Secondary objectives are to determine specified comparative clinical and cost-effectiveness outcomes, and to explore, in a linked qualitative study, the lived experiences of patients with hip OA and experiences and impact of, ultrasound-guided intra-articular hip injection.

**Methods:**

The HIT trial is a pragmatic, three-parallel group, single-blind, superiority, randomised controlled trial in patients with painful hip OA with a linked qualitative study. The current protocol is described, in addition to details and rationale for amendments since trial registration. 204 patients with moderate-to-severe hip OA will be recruited. Participants are randomised on an equal basis (1:1:1 ratio) to one of three interventions: (1) best current treatment, (2) best current treatment plus ultrasound-guided intra-articular hip injection of corticosteroid (triamcinolone acetonide 40 mg) with 1% lidocaine hydrochloride, or (3) best current treatment plus an ultrasound-guided intra-articular hip injection of 1% lidocaine hydrochloride alone. The primary endpoint is patient-reported hip pain intensity across 2 weeks, 2 months, 4 months and 6 months post-randomisation. Recruitment is over 29 months with a 6-month follow-up period. To address the primary objective, the analysis will compare participants’ ‘average’ follow-up pain NRS scores, based on a random effects linear repeated-measures model. Data on adverse events are collected and reported in accordance with national guidance and reviewed by external monitoring committees. Individual semi-structured interviews are being conducted with up to 30 trial participants across all three arms of the trial.

**Discussion:**

To ensure healthcare services improve outcomes for patients, we need to ensure there is a robust and appropriate evidence-base to support clinical decision making. The HIT trial will answer important questions regarding the clinical and cost-effectiveness of intra-articular corticosteroid injections.

**Trial registration:**

ISRCTN: 50550256, 28th July 2015.

## Background

Hip osteoarthritis affects a substantial, and growing, number of people worldwide. Its incidence and prevalence are increasing due to ageing and obesity. It is estimated that between 10 and 18% of those aged over 60 years are affected, rising to one in three patients over the age of 85 years [1], with a substantial proportion experiencing persistent pain, loss of function and decline in health-related quality of life [2].

In 2016, in the United Kingdom (UK), an estimated 92,465 primary total hip replacement (THR) operations were carried out, with approximately 90% being performed for OA [3]. Whilst not all patients with hip osteoarthritis will need a THR, the numbers of THR are continuing to rise [4, 5]. Patients with hip OA are typically treated in primary care for several years before referral for surgical opinion, with evidence suggesting that primary care management is suboptimal and that patients with hip pain feel their pain is neglected despite consulting their general practitioner (GP) [6]. In one observational study from the Netherlands, patients with incident hip OA remained under GP care for an average of 7 years (82 months) before referral to orthopaedics, suggesting that a considerable period of time is available to help patients through the application of non-surgical interventions before surgery is suggested as an option [7]. This is important, as non-surgical interventions could significantly contribute to postponing hip replacement [8], which in turn can delay or prevent future revision surgery.

A range of treatments, including analgesia and exercise therapy, are available to help people with hip OA, although the evidence supporting their use, especially in primary care settings, is limited. Influential organisations including the European League Against Rheumatism (EULAR), Arthritis Research UK and the National Institute for Health and Care Excellence (NICE) highlight the lack of evidence from randomised controlled trials (RCTs) conducted exclusively with people with hip OA [9, 10]. NICE guidance for the non-surgical treatment of OA advises a combination of non-pharmacological and pharmacological treatments, with education, exercise and weight reduction being core treatments [9]. Options for analgesia include paracetamol, oral non-steroidal anti-inflammatory drugs (NSAIDs) and opiates, with intra-articular corticosteroid injection recommended as an adjunct for those with moderate-severe pain.

Intra-articular corticosteroid injection is not used to treat patients with hip OA as widely as it could be in the UK owing to a lack of local availability and uncertainties about patient selection and potential benefit [11]. Intra-articular hip injection is typically administered using imaging guidance, either fluoroscopy or ultrasound, to improve the accuracy of the injection [12]. The evidence supporting the use of intra-articular corticosteroid injection for hip OA is limited and conflicting. Of the five published RCTs of intra-articular corticosteroid injection for hip OA [11, 13–16], all recruited small numbers of participants from secondary care (≤40 per treatment group) and employed short-term follow-up (maximum three months). Whereas two RCTs with hip OA patients have demonstrated clinical benefits at eight weeks post-injection [11, 16] and two at three months [14, 16], one reported no significant difference in pain or function at three months [15]. There is evidence that beneficial effects of knee injections persist to six months [17]. All previous RCTs of corticosteroid injections for hip OA have included a ‘placebo’ arm of injections of either local anaesthetic or saline but only one has compared clinical effectiveness of corticosteroid injection with best usual care [11].

There is, therefore, promising evidence to support the use of intra-articular corticosteroid injection for patients with hip OA, although current research is restricted to small studies of more severely affected patients with only short-term follow-up. The available studies therefore do not reflect the true range of disease managed in primary care, where patients may have less severe disease and are more likely to have multi-morbidity which limits their other treatment options. To ensure healthcare services improve outcomes for patients we need to ensure there is a robust and appropriate evidence-base to support clinical decision making.

## Objectives

The current protocol (version 6.0, 3 April 2018) is described below. Key amendments to the protocol since trial registration are highlighted with comments in square brackets and described in detail, along with rationale for change, in Table 1.

**Table 1:** Key Protocol Amendments since Trial registration

Protocol Section	Original protocol	Amended details	Date of Amendment REC approval	Rationale for amendment
Secondary objectives	To explore reasons for non-participation in the study and perceptions of recruitment processes with aim of identifying any modifiable barriers to recruitment [To undertake only if recruitment less than anticipated at 3 month review]	Objective removed	May 2018	We could not to complete the qualitative objective - to explore reasons for non-adherence - due to low recruitment to this qualitative study of people who were eligible for the trial but unwilling to participate. Furthermore, we had identified key modifiable issues to facilitate method of recruitment relating to eligibility criteria (see below) and recruitment route (see ‘Progress of the trial’).
Inclusion Criteria	Moderate-to-severe hip pain (a score of four or more on a 0–10 numeric rating scale (NRS)) on the day of assessment	Moderate-to-severe hip pain (a score of four or more on a 0–10 numeric rating scale (NRS)) on average over the last 2 weeks and current hip pain rated as at least 1 out of 10 (on a 0–10 NRS) on the day of assessment	September 2016	During the first 5 months of recruitment we observed that, due to the day-to day variability of osteoarthritis symptoms, a number of potential participants did not meet the eligibility criterion of pain of 4/10 on the day of assessment.
Exclusion Criteria	Any contraindications to the use of 1% lidocaine hydrochloride as listed in Summary of Product Characteristics (SPC) e.g. complete heart block, hypovolaemia,	Any contraindications to the use of 1% lidocaine hydrochloride as listed in Summary of Product Characteristics (SPC) e.g. complete heart block, hypovolaemia, porphyria	September 2016	A review of the summary of product characteristics (SPC) for lidocaine hydrochloride revealed a new contraindication (porphyria). Porphyria was added to the exclusion criteria as part of an urgent safety measure and subsequent amendment.
	Receiving anticoagulants (warfarin, dabigatran, rivaroxaban, apixaban or low molecular weight heparin)	Receiving anticoagulants (warfarin, dabigatran, rivaroxaban, apixaban or low molecular weight heparin), ritonavir or cobicistat	January 2017	It was noted in the MHRA Drug Safety Update dated 14 December 2016 ‘Cobicistat, ritonavir and coadministration with a steroid: risk of systemic corticosteroid adverse effects’ that coadministration of corticosteroids, including intra-articular triamcinolone, with an HIV-treatment-boosting agent may increase the risk of corticosteroid side-effects including adrenal insufficiency, adrenal suppression and Cushing’s syndrome, due to a pharmacokinetic interaction. Receiving cobicistat or ritonavir were added to the exclusion criteria as part of an urgent safety measure and subsequent amendment.
Sample Size	To address the primary objective, the analysis will be based on comparisons of participants’ ‘average’ follow-up pain NRS scores, based on a random effects linear repeated-measures model, with a ‘cluster’ size of 4 (denoting 4 follow-up assessments) and a postulated coefficient of 0.5. A sample size of 232 will provide 90% power (5% two-tailed significance) to detect a minimum difference of 1.5 points in mean pain NRS score (anticipated baseline SD of pain scores = 4.5 points; effect size of 0.33) between I1 and I2 across the 6-month follow-up period, allowing for total of 15% loss to follow-up. As our trial also evaluates I3 (against I1), we have three groups of interest and hence need 348 participants.	To address the primary objective, the analysis will be based on comparisons of participants’ ‘average’ follow-up pain NRS scores, based on a random effects linear repeated-measures model, with four follow-ups and postulated correlations of 0.5 for repeat-measures and 0.2 for baseline-outcome. A sample size of 136 (68 per arm) provides 80% power (5% two-tailed significance) to detect a minimum difference of 1 point in mean pain NRS score (anticipated SD of about 2.5; effect size of 0.4) between I1 and I2 across the 6-month follow-up period, allowing for 15% loss to follow-up. As the trial also evaluates I3 (against I1), there are three groups of interest and hence 204 participants are needed.	May 2018	The Data Monitoring Committee noted poor recruitment and suggested rerunning the sample size calculations to ensure the original sample size assumptions were still valid. The observed baseline standard deviation (SD) of the primary outcome based on data collected from participants recruited by this time point (n = 65) was 1.7 (and the SD for follow up scores was around 2.5) – i.e. much lower than the SD of 4.5 expected before the start of recruitment on which the original sample size calculation was based. The clinically important difference of 1.5 (originally stated) in the context of this baseline SD would be ‘large’ (effect size above 0.8). The clinically important difference of 1.5 was considered to be too large in relation to the lower expected SD. The clinically important difference for the NRS-pain scale has taken different values across studies; an absolute difference of 1 has been specified in some studies (which would relate to a “moderate” effect size (0.5) when the SD is around 2; or, 0.4 in relation to higher SD of 2.5 which is observed across follow up time points). Hence, we felt that a revised effect size of 0.4 is justifiable. Using this revised effect size of 0.4 and revised power of 80% (on the advice of the TSC), the sample size was amended as described, and was approved by the funder, TMC and DMC.
Recruitment period	The monthly target for recruitment will be 20. A recruitment period of 18 months will therefore be required.	An amended recruitment period of 29 months is required to meet the revised sample size (*n* = 204). The monthly target for recruitment has been adjusted from 20 to 10.	May 2018	During the first 18 months of trial recruitment an average of 7 patients per month were recruited. Recruitment improved since greater emphasis has been placed on recruitment route 3 to an average of 10 per month. Recruitment period was recalculated based on observed recruitment and revised sample size.

### Primary objective

The primary objective of this trial is:

- To compare longitudinal pain scores over 6 months, in people with hip OA, between those receiving an ultrasound-guided intra-articular hip injection of corticosteroid with 1% lidocaine hydrochloride plus best current treatment with those receiving best current treatment alone.

### Secondary objectives

The secondary objectives of this trial are:

- To compare the clinical effectiveness of an ultrasound-guided intra-articular hip injection of corticosteroid and 1% lidocaine hydrochloride and best current treatment with best current treatment alone across a range of secondary outcome measures including physical function, stiffness, patient global impression of change, general health, sleep, self-efficacy, and satisfaction with treatment
- To compare the effect of an ultrasound-guided injection of corticosteroid and 1% lidocaine hydrochloride plus best current treatment with an ultrasound-guided injection of 1% lidocaine hydrochloride plus best current treatment on pain, physical function, stiffness, patient global impression of change, general health, sleep, self-efficacy, and satisfaction with treatment
- To compare the cost-effectiveness of ultrasound-guided intra-articular injection of corticosteroid and 1% lidocaine hydrochloride and best current treatment with best current treatment alone over 6 months
- To explore in a linked qualitative study, the acceptability to, and impact of, ultrasound-guided intra-articular joint injection to patients with hip OA
- To explore in a linked qualitative study, the experiences of patients of living with hip OA
- To explore reasons for non-participation in the study and perceptions of recruitment processes with aim of identifying any modifiable barriers to recruitment (to undertake only if recruitment less than anticipated at 3 month review). [Amended, see Table 1]

## Methods

### Trial design

The trial is a pragmatic, three-parallel group, single-blind, superiority, randomised controlled trial in patients with painful hip OA. The intervention arms are:

- best current treatment (intervention arm 1, I1)
- best current treatment plus ultrasound-guided intra-articular hip injection of corticosteroid (triamcinolone acetonide 40 mg) and 1% lidocaine hydrochloride (intervention arm 2, I2)
- best current treatment plus ultrasound-guided intra-articular hip injection of 1% lidocaine hydrochloride alone (intervention arm 3, I3)

Participants with moderate-to-severe hip OA consulting at musculoskeletal clinics at the primary-secondary care interface and in secondary care or identified from a Read Code search at participating General Practices are randomised on an equal basis (1:1:1 ratio) to one of these intervention arms. Follow-up data are gathered from participants at 2 weeks, 2 months, 4 months and 6 months via postal questionnaires. The experience and impact and perceptions of receiving an intra-articular hip injection for hip OA compared to best current treatment alone are explored in in-depth qualitative interviews in a purposive sample of approximately 30 participants (*n* = 10 from each of the three arms of the trial).

### Study setting

Participants are recruited from primary care referrals to orthopaedics, rheumatology and two musculoskeletal National Health Service (NHS) interface services in Staffordshire, UK. These services are variously staffed by GPs with specialist musculoskeletal interests, extended-scope physiotherapists, rehabilitation medicine specialists, rheumatologists and orthopaedic surgeons. The musculoskeletal hip clinics, where patients are screened, consented and treated, take place at the two musculoskeletal interface services in Staffordshire.

The research sites received local management approval, trial specific training covering the interventions (with competency in delivery of the trial interventions being confirmed) and trial administrative procedures, and undertook sufficient research preparation (including Good Clinical Practice (GCP) training) prior to the start of recruitment into the trial.

### Participants

The study population consists of participants with moderate-severe pain attributable to hip OA. Diagnosis is based on presenting symptoms and routine clinical history and examination supported by radiographic evidence of hip OA.

#### Inclusion criteria

1. Male or female aged ≥40 years
2. A clinical diagnosis of unilateral or bilateral hip OA, and confirmed on plain radiography within the last 24 months
3. Moderate-to-severe hip pain (a score of four or more on a 0–10 numeric rating scale (NRS)) on average over the last 2 weeks and current hip pain rated as at least 1 out of 10 (on a 0–10 NRS) on the day of assessment [amended, see Table 1]
4. Symptom duration of episode of at least 6 weeks
5. Hip pain occurring on most days of the last month [18]
6. Informed written consent provided by the patient

#### Exclusion criteria

1. Hip pain due to other disorders (e.g. trochanteric bursitis, avascular necrosis, pain referred from back).

2. Intra-articular corticosteroid injection into the affected hip or ipsilateral trochanteric bursa injection within the preceding 3 months.

3. Any previous surgery on the affected hip.

4. Clinical suspicion of local or systemic sepsis or infection.

5. Current or previous infection of the affected hip.

6. Significant trauma to the affected hip requiring immobilisation in the previous 3 months.

7. Unwillingness to undergo study interventions.

8. Unable to understand and complete self-report questionnaires written (or spoken) in English.

9. Significant illness (known or suspected) including, but not limited to:

• inflammatory joint disease (e.g. rheumatoid arthritis, seronegative spondyloarthropathy (ankylosing spondylitis, psoriatic arthritis, reactive arthritis, inflammatory-bowel disease associated inflammatory arthritis)).

• polymyalgia rheumatica or other condition requiring regular oral steroid use.

• malignancy (where malignancy is thought to be causing hip pain e.g. suspected bony metastases).

• any other severe medical illness which in the opinion of the local Principal Investigator (PI)(or other authorised clinical delegate) precludes trial participation.

10. Pregnant or lactating females.

11. Receiving anticoagulants (warfarin, dabigatran, rivaroxaban, apixaban or low molecular weight heparin), ritonavir or cobicistat [Amended, see Table 1].

12. Any history of hypersensitivity to triamcinolone acetonide or 1% lidocaine hydrochloride or any of their excipients (1 N Hydrochloric Acid QS, 1 N Sodium Hydroxide QS, Benzyl alcohol. Polysorbate 80, Sodium carboxymethylcellulose and Sodium chloride).

13. Any contraindications to the use of 1% lidocaine hydrochloride as listed in Summary of Product Characteristics (SPC) e.g. complete heart block, hypovolaemia, porphyria [Amended, see Table 1].

#### Patient identification

Potential participants are identified via three routes:

1. GP referrals of patients with hip pain into participating NHS musculoskeletal services at two locations. Local GPs are informed that the trial is taking place and encouraged to refer patients whom they feel may be eligible to participate. Pop-up electronic reminders are incorporated into GP electronic record systems to remind GPs if they enter a code for hip pain or OA during a primary care consultation. Patients are triaged into dedicated musculoskeletal hip research clinics and are sent the Participant Information Leaflet (PIL) prior to their appointment.
2. Patients who have not been directly booked or triaged into the musculoskeletal hip clinic, but who have been referred for management of hip OA to other musculoskeletal, rheumatology and orthopaedics clinics are also identified at their initial appointment. Those deemed to be eligible and interested are given a PIL by the treating clinician.
3. The electronic records of GP practices in the local clinical research network are searched to identify patients consulting with hip pain in the last 12 months. This search is conducted periodically, within small groups of local practices in order to invite consulters with hip pain to the musculoskeletal hip clinic. The search will generate a list of NHS numbers which are screened so that those individuals who have already been referred to the musculoskeletal hip clinic can be excluded. Those who have not already been referred into the service are sent a PIL and a letter inviting them to telephone the Keele Clinical Trials Unit (CTU) if they are interested in attending the musculoskeletal hip clinic. Those who telephone the CTU administrator will receive a brief, provisional eligibility screening. Those who are eligible at this stage are made an appointment for the musculoskeletal hip clinic.

#### Participant screening

Patients identified by routes 1 and 2 above will attend for their routine clinic appointment, according to normal NHS clinical attendance procedures. For patients identified by route 3, a brief, provisional eligibility screen will have been carried out over the telephone by the CTU administrator prior to the appointment being made and a preliminary informed consent to ‘screening assessment’ are taken prior to any eligibility assessment.

The clinical consultation is undertaken by the local PI (or authorised delegate). In accordance with the UK Clinical Trial Regulations, a patient’s eligibility to participate in the trial is the responsibility of a medically qualified doctor. All patients presenting with painful hip OA are considered for inclusion and an Eligibility Screening Form completed.

Patients who have not had a hip radiograph in the past 24 months will undergo radiography as part of eligibility screening. For patients identified via routes 1 and 2 this is in line with usual clinical practice and normal procedures in the musculoskeletal clinic. For patients identified via route 3, radiographs are obtained as a study procedure if they have not had a radiograph performed in the last 24 months. The preliminary consent to ‘screening assessment’ is performed prior to these radiographs being obtained.

Anteroposterior (AP) pelvis and lateral oblique views are obtained as detailed below.

AP pelvis: The patient lies supine on the table with their legs extended and their head resting on a pillow. The median sagittal plane (MSP) is at 90 degrees to the table top and the anterior superior iliac spines (ASISs) are at equal distance from the table-top. The arms are raised onto the pillow. The legs are slightly internally rotated to bring the necks of femora parallel to the table-top. Gonad protection is applied if appropriate. The beam is centred in the midline, midway between the ASIS and the upper border of the symphysis pubis.

Lateral oblique hip: From the initial AP pelvis position the patient is rotated laterally through 45 degrees onto the side under examination and supported in this position with foam pads. The knee and hip are flexed and externally rotated to bring the lateral aspect of the thigh in contact with the table-top. The arms are rested on the pillow. Gonad protection is applied. The beam is centred to the femoral pulse.

#### Recruitment and consent

Eligible patients who are interested in trial participation are invited by the local PI (or authorised delegate) to see a researcher or research assistant who will explain the trial in full. The researcher or research assistant will have received appropriate training and be authorised on the site trial delegation log. Patients are able to ask questions about study involvement. Those who remain interested in participation after seeing the researcher are consented by a researcher, complete baseline data collection, undergo randomisation and receive the intervention during the same clinic visit.

Documented reasons for ineligibility or declining participation are monitored by the Keele CTU as part of a regular review of recruitment progress.

Patients who decline assessment of eligibility, who are either ineligible to participate or who do not wish to participate are thanked for their attendance and instructed to consult their GP should their symptoms continue (route 3) or managed as per usual care (routes 1 and 2).

### Randomisation

#### Allocation

Participants are randomised by an administrator in clinic after consent, baseline data collection and delivery of best current treatment in a 1:1:1 ratio, via the Keele CTU’s web-based randomisation service. This is a secure web-based randomisation system with emergency telephone back-up. The randomisation sequence is computer-generated. Once randomised, the authorised staff member is notified of the participant’s treatment allocation.

#### Allocation concealment

Concealment of the allocation process is ensured through the remote computer-generation of the randomisation sequence and web-based interface including entry of participant details and necessary consent prior to a unique participant identification number being generated and disclosure of treatment allocation.

#### Sequence generation

Blocking (of individual participants) is used as the unit randomisation method to ensure similar numbers of participants are allocated to the three treatment arms. Random permuted block sizes of 3 and 6 are used to give 96 possible randomisation sequences (for each unit of randomisation) with equal chance (determined using a computer-generated random function). The sequence is held within the computer system and not known to researchers/administrators so as to preserve concealment integrity, and hence comparability of participant allocation between study arms.

#### Blinding

Participants and clinicians will not be blind to allocation to best current treatment only (I1) or injection (I2 or I3). However, for those participants randomised to either of the two injection arms, participants are blind to the exact nature of the injection (triamcinolone acetonide plus 1% lidocaine hydrochloride or 1% lidocaine hydrochloride alone) to ensure the validity of the 1% lidocaine hydrochloride injection as a credible placebo. The Research Nurse will remain blind to treatment allocation to enable the nurse who conducts Minimum Data Collection (MDC) to remain unaware of allocation. The statistician will also be blind to treatment allocation. The qualitative researcher is blind to injection group allocation.

Those injected are informed to carry a trial information card at all times during the first 2 months and to present it to medical staff should they be admitted to hospital during their participatory period (6 months). 24-h emergency unblinding is available.

### Interventions

The interventions are delivered in the context of a ‘one-stop’ clinic where assessment, baseline data collection, randomisation and intervention all occur within the same visit.

If bilateral symptoms are present, the hip with the most severe symptoms according to the participant is treated. In the event that both hips are equally affected, the participant is asked to choose which hip to treat. The contralateral hip may be treated in accordance with local guidelines (excluding intra-articular hip injection). Any treatment to the contralateral hip does not constitute trial treatment and is captured through the participant follow-up questionnaires.

#### Intervention arm 1 (I1): Best current treatment

Participants randomised to this intervention receive written information (the Arthritis Research UK Osteoarthritis leaflet [19] and a bespoke HIT trial leaflet on exercise and functional activities), and personalised advice and information about weight loss, exercise, footwear, walking aids and optimising pain management, delivered by the PI (or authorised delegate) within the clinic visit.

#### Intervention arm 2 (I2): Best current treatment plus intra-articular injection of corticosteroid plus lidocaine

Participants randomised to this intervention receive best current treatment as I1 plus one ultrasound-guided intra-articular injection of 40 mg triamcinolone acetonide and 4mls 1% lidocaine hydrochloride into the hip.

Both triamcinolone acetonide 40 mg/ml sterile, aqueous suspension and 1% lidocaine hydrochloride 10 mg/ml solution for injection are prepared and handled in line with manufacturer’s recommendations as outlined and in accordance with the SPC.

A disposable 25G needle and syringe is be used for regional anaesthesia of the skin and superficial soft tissues and a 22G spinal needle and syringe is used for regional anaethesia of deeper soft tissues and intra-articular injection.

The following technique is observed when applying intra-articular injections:

Preparation for the injection includes: LOGIQ ultrasound system with a 1-4 MHz curvilinear transducer (GE Healthcare, Hatfield, England); disposable sterile transducer sheath; disposable 25G needle for regional anaesthesia of skin and superficial soft tissues, and disposable 22G spinal needle for regional anaesthesia of deeper soft tissues and intra-articular injection. Three syringes are prepared containing: 3mls 1% lidocaine hydrochloride; 4mls 1% lidocaine hydrochloride and 1 ml 40 mg triamcinolone acetonide.

A sterile aseptic technique is observed. The participant lies supine with legs extended into a neutral position of comfort. The skin is cleaned with chlorhexidine 0.5% solution. The transducer is covered with gel and a sterile sheath. Sterile gel is applied to the external surface of the sheath. The anterior capsule of the hip joint is located using ultrasound guidance and 3mls 1% lidocaine hydrochloride is introduced to the overlying skin and superficial soft tissues using a 25 g needle. A 22G spinal needle will then be inserted, observing its route in real-time by ultrasound, until its tip is seen to enter the anterior joint capsule. 1 ml of 1% lidocaine hydrochloride is injected into the hip to confirm correct placement and 40 mg of triamcinolone acetonide (1 ml volume) with a further 3mls of 1% lidocaine hydrochloride is injected showing distension of the capsule by the fluid under ultrasound (total intracapsular volume 5mls). The needle is withdrawn, haemostasis ensured and a dressing applied over the injection site. Participant should be advised to wait 15 min following injection or alternatively ensure that they are accompanied by a responsible adult for that time. Participants are advised to observe minimal weight-bearing for 24 h following the injection, and not to drive for 24 h.

It is understood that there may be slight variations to the described technique due to previous training. All injectors will undergo trial-specific training in injection technique and there is a standard operating procedure in place.

#### Intervention arm 3 (I3): Best current treatment plus intra-articular injection of lidocaine

Participants randomised to this intervention will receive one ultrasound guided intra-articular injection of 5mls 1% lidocaine hydrochloride into the hip plus best current treatment.

The injection procedure is conducted as described above (I2) with two exceptions. Three syringes are prepared containing: 3mls 1% lidocaine hydrochloride; 4mls 1% lidocaine hydrochloride and 1mls 1% lidocaine hydrochloride. 1 ml of 1% lidocaine hydrochloride is injected into the hip to confirm correct placement and a further 4mls of 1% lidocaine hydrochloride is then injected showing distension of the capsule by the fluid under ultrasound (total intracapsular volume 5mls).

Injections are delivered by clinicians (2 Consultant rheumatologists, 2 Extended Scope Physiotherapists and one Consultant musculoskeletal sonographer) who are fully trained in the technique and work in the musculoskeletal services. Since musculoskeletal ultrasound is highly operator-dependent and relies on robust training with direct supervision to gain clinical competency, clinicians performing US-guided injections have extensive clinical experience performing US guided injections and will have their competency assessed by a Consultant musculoskeletal sonographer prior to commencement of the trial.

### Crossover and post-trial care

Participants cannot cross over from one arm of the trial to the other. If a participant declines injection after randomisation, this is recorded as a protocol deviation and the participant’s data included in the Intention-to-Treat (ITT) analysis. Similarly, if a participant in the best current treatment arm (I1) of the trial receives an injection as part of standard care they are managed as a protocol deviation and included in ITT analysis.

Participants’ clinical care after the ‘one-stop’ research clinic returns to usual NHS healthcare.

### Outcomes

The primary endpoint is patient-reported hip pain intensity over the whole of 6-months follow-up (through repeated measures at 2 weeks, 2 months, 4 months and 6 months) post-randomisation).

Secondary endpoints are at each of the individual follow-up points (i.e. 2 weeks, 2 months, 4 months and 6 months post-randomisation).

#### Primary outcome measure

Patient-reported hip pain intensity measured using a 0–10 Pain Numeric Rating Scale for current hip pain (hip pain today) [20].

#### Secondary outcome measures

The secondary outcomes include pain, stiffness and everyday physical function (Western Ontario and McMaster University Arthritis Index (WOMAC v 3.1)) [21], participants’ self-reported global impression of change [22], general health (SF-12, EQ-5D-5 L) [23, 24], sleep disturbance (adapted from Dawson et al. [25]), pain self-efficacy [26], illness perceptions [27], maintenance of and return to desired activities including work and social life, healthcare utilisation including medication use and participant incurred cost, treatments received (including referral for surgery and analgesia), participants’ satisfaction with treatment, work (employment status, performance, absence), joint replacement surgery (National Joint Register). Table 2 summarises the content of the participant questionnaires.

**Table 2:** Participant Questionnaire Content

Visits	Baseline	2-week	2-month	4-month	6-month
Baseline measures					
Demographics (date of birth, gender)	✓	✓	✓	✓	✓
Demographics (marital status)	✓				
Hip pain questions: uni/bilateral, duration	✓				
Previous hip injection	✓				
Participant treatment preference and expectations	✓				
Comorbidity	✓				
Self-reported height and weight (BMI)	✓				✓
Other musculoskeletal pain (Body manikin) [22]	✓				
Anxiety and Depression – GAD and PHQ8 [23, 24]	✓				
Primary Outcome measure					
Pain - Numerical Rating Scale score for current pain [20]	✓	✓	✓	✓	✓
Secondary outcome measures					
Function - Western Ontario and McMaster University Arthritis Index (WOMAC v3.1) [32]	✓		✓	✓	✓
Participant’ self-reported global impression of change [33]		✓	✓	✓	✓
General health (SF-12) [34]	✓		✓	✓	✓
Sleep disturbance (Likert type scale, adapted from Dawson et al. [35])	✓		✓	✓	✓
Pain self-efficacy [36]	✓		✓	✓	✓
Modified Brief Illness Perceptions Questionnaire [37]	✓		✓		✓
Satisfaction and experience			✓		✓
Health Economic Outcomes					
Health status - EQ5D-5 L [38]	✓	✓	✓	✓	✓
Employment status	✓				✓
Performance at work	✓		✓		✓
Absenteeism from work	✓		✓		✓
Health care utilisation			✓		✓
Participant -incurred costs			✓		✓
Process Data					
Other hip injections received			✓		✓
Self-reported adverse events		✓	✓		
Adherence to best current treatment advice			✓	✓	✓

### Adverse events

Triamcinolone acetonide and 1% lidocaine hydrochloride have specific licenses for the treatment of osteoarthrosis (osteoarthritis) and regional anaesthesia specifically and have been widely used for many years in standard practice in both primary and secondary care and have very well-established and understood safety profiles [21]. They are being used in accordance with the guidance given in the British National Formulary (BNF) and Map of Medicine guidance for injection in hip OA [9, 10]. The incidence of adverse predictable undesirable side-effects associated with the use of corticosteroids correlates with the relative potency of the drug, dosage, and timing of administration and duration of treatment, and therefore based on the dosage to be used in this study, there is no requirement to record non-serious adverse events beyond normal clinical practice. The following events will not be recorded as Serious Adverse Events (SAEs) within this trial:

Hospitalisation for:

- Routine treatment or monitoring of hip OA associated with any deterioration in condition
- Treatment which is elective or pre-planned, for a pre-existing condition not associated with any deterioration in condition
- Prolongation of hospitalisation not associated with an adverse event
- Admission to hospital or other institution for general care, not associated with any deterioration in condition
- Treatment on an emergency, outpatient basis for an event not fulfilling any of the definitions for serious as given above and not resulting in hospital admission

Where a SAE or Suspected Unexpected Serious Adverse Reaction (SUSAR) occurs, reporting procedures are in place that are in accordance with good clinical practice guidance and the requirements specified by the Medicines and Healthcare products Regulatory Agency (MHRA). All SAEs are considered by the external monitoring committees.

### Data collection

All participants enrolled in the trial are asked to complete a paper questionnaire at the baseline clinic appointment and a posted questionnaire after 2 weeks, 2 months, 4 months and 6 months. Questionnaires will capture data on all outcome measures (Table 2).

Baseline data collection includes: demographics (date of birth, gender, marital status); hip pain questions: uni/bilateral, duration; previous hip injection; participant treatment preference and expectations; comorbidity; self-reported height and weight (Body Mass Index - BMI), other musculoskeletal pain (Body manikin) [22] and anxiety and depression – Generalised Anxiety Disorder (GAD) and Patient Health Questionnaire 8 [23, 24].

For participants receiving I2 and I3, bilateral ultrasound images are stored, and scored on the presence or absence of synovitis and effusion. Participants are also asked which injection they think they received.

Non-responders to the 2-week follow-up postal questionnaire are sent a repeat questionnaire and PIL after 10 calendar days. Those who do not respond to the repeat questionnaire within 10 days are telephoned by the Research Nurse (who will remain blind to group allocation), in order to try to capture key primary outcome data and to minimise missing data. A postal MDC form is mailed to the participant if the participant has not been contacted after 5 phone-call attempts.

At other follow-up time-points, non-responders are sent a reminder postcard after 10 days. Those who do not respond to the reminder postcard are sent a repeat questionnaire and Participant Information Sheet with a further covering letter after a further 10 days. Non-responders to the repeat questionnaire are telephoned by the Research Nurse (blind to treatment allocation) 10 days later. A postal MDC form are mailed to the participant if the participant has not been contacted after 5 phone-call attempts.

**Fig. 1:**
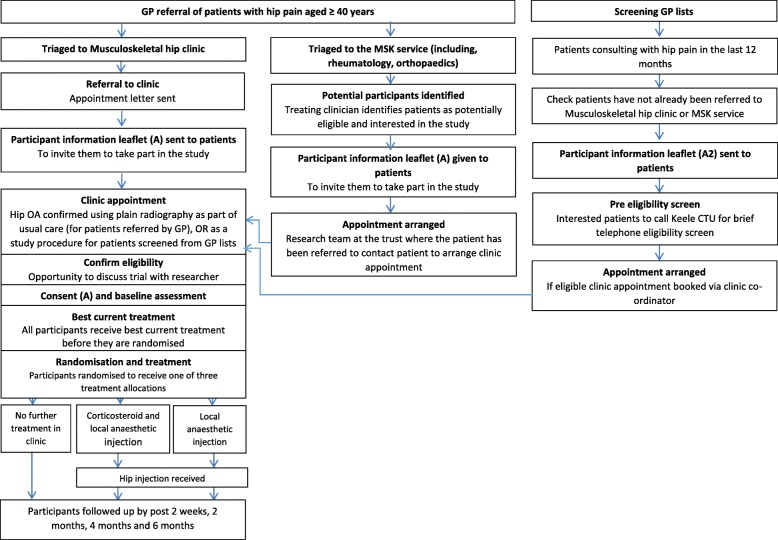
Flow of participants through trial

The flow of events as participants proceed through the trial is outlined in (Fig. 1) and the timing of key events outlined in Table 3.

**Table 3:** Participant Timeline

Visits	Pre-Baseline	Baseline Day 0	Randomisation Day 0	Intervention Day 0	2-week follow-up	2-month follow-up	4 month follow-up	6-month follow-up
				Duration of Intervention				
Events								
Brief telephone screening (identified via route 3)	✓							
Consent to eligibility screening assessment (route 3)		✓						
Eligibility Screening		✓						
Full informed consent by researcher/ research assistant		✓						
Randomisation			✓					
Participant Questionnaire (see Table 1)		✓			✓	✓	✓	✓
Web-based randomisation			✓					
Administration of study intervention				✓				
SAE reporting				✓	✓	✓	✓	✓
Expedited reporting (< 24 h) of Suspected Unexpected Serious Adverse Reactions (SUSARs)				On going				

### Sample size

To address the objectives of the trial, 204 participants (68 in each arm) need to be recruited over a 29-month period [amended since trial registration, see Table 1 and ‘Progress of trial’]. To address the primary objective, the analysis will be based on comparisons of participants’ ‘average’ follow-up pain NRS scores, based on a random effects linear repeated-measures model, with four follow-ups and postulated correlations of 0.5 for repeat-measures and 0.2 for baseline-outcome. A sample size of 136 (68 per arm) provides 80% power (5% two-tailed significance) to detect a minimum difference of 1 point in mean pain NRS score (anticipated SD of about 2.5; effect size of 0.4) between I1 and I2 across the 6-month follow-up period, allowing for 15% loss to follow-up. As the trial also evaluates I3 (against I1), there are three groups of interest and hence 204 participants are needed.

### Statistical methods

The analysis will be undertaken according to an analysis plan agreed with the TSC and DMC, incorporating statistical analysis, health economics and qualitative analysis.

The main statistical analysis is based on reporting guidelines for the design and conduct of parallel-arm trials [25]. The main treatment analysis will be conducted blinded to treatment allocation and will be analysed on an intention to treat (ITT) approach with all randomised participants retaining their original randomised group. Estimation through linear mixed modelling takes account of missing data (under the missing at random (MAR) missingness assumption). Analysis will be adjusted for baseline covariates (pain, age and gender). Additional sensitivity analysis will address the robustness of the findings to deviation from the MAR assumption – specifically through considering plausible ‘missing not at random’ (MNAR) scenarios.

Key baseline characteristics of participants in the three treatment groups will be presented to illustrate comparability. The linear mixed model will be used to derive estimates of ‘average’ pain across the four time points (thus reflecting summary pain assessment across the full follow-up period) as well as estimates of pain at each time point (by modelling the interaction of group by time). In each evaluation, a random-slope model will be preferred (to a random-intercept model) if the goodness-of-fit of the model is significantly improved through time-projected random-slope specification. Statistical significance is at the 5% probability level (two-tailed). The collection of data and statistical analysis will be performed blinded to treatment allocation. The primary clinical evaluation will be a comparison of pain NRS scores over the 6-month follow-up period (with repeated measures at 2 weeks and 2, 4 and 6 months) between those treated with best current treatment plus corticosteroid and lidocaine injection, and those treated with best current treatment alone.

The secondary clinical evaluations will include: evaluation of pain NRS score differences between groups at each of the individual time points (2 weeks, 2 months, 4 months and 6 months) as well as between-group comparison of secondary outcome measures over the 6-month follow-up period. Mixed models for repeated measures (MMRM) will be through linear-regression in respect of numerical outcomes, and logistic-regression in relation to categorical outcome measures. A number of follow-up data are anticipated to be retrieved through minimum data collection, and therefore we will use multiple imputation (MI) using chain equations [26] to impute missing secondary outcome data – the prediction models will include baseline variables and the (primary) pain outcome measure as predictors. MI, similar to mixed modelling, estimates effect on the basis of an MAR missingness mechanism. Estimation of the primary outcome measure based on MI evaluation will be carried out as a sensitivity analysis of the main MMRM analysis. In addition, an MI analysis incorporating plausible deviations from ignorable missingness will be considered in respect of sensitivity analysis of the primary outcome to MNAR missing data.

The primary analyses will be adjusted for the following baseline covariates: pain score, age and gender (and EQ5D for health economic evaluation). Estimates of clinical effect will be shown as mean (SDs) differences (for numerical outcomes) and odds ratios (for dichotomous outcomes), with 95% confidence intervals (CI). As previously stated, regression analysis will be based on a linear model for numerical outcomes and binary logistic model for dichotomous outcomes with a random-effects component being added to the model for repeated measures data, to take into account the intra-cluster correlation. To help in interpreting the size of the estimated clinical effect for the between-group difference in pain (overall and at individual time points) we will calculate the standardised mean difference (‘effect size’) which is the ratio of the estimated mean difference to the standard deviation of pain scores in the total randomised population. For all evaluations of the primary outcome, we will present mean and 95% confidence interval estimates. The number needed to treat (NNT) with 95% CI will be calculated in respect of the comparison between best current treatment plus corticosteroid and 1% lidocaine hydrochloride injection and best current treatment alone [27].

Exploratory subgroup analysis will include evaluation of variables such as

- Participants’ expectations regarding treatment response
- Participants’ treatment preference
- Illness perceptions
- Adherence to best current treatment
- Presence of synovitis or effusion on ultrasound
- BMI
- Duration of symptoms
- Severity of symptoms

### Economic evaluation

The health economic analysis will determine the cost-effectiveness of intra-articular corticosteroid injection for hip OA and best current treatment compared with best current treatment alone. A cost-consequence analysis will initially be reported, describing all important results relating to costs and outcomes. An incremental cost-utility analysis will be undertaken using participant responses to the EuroQoL EQ-5D-5 L questionnaire at baseline and each follow-up time-point, to calculate the cost per additional Quality-Adjusted Life Year (QALY) gained. The base-case analysis will adopt an NHS perspective and will include costs of the intervention and best current treatment, and other hip OA-specific health care utilisation. These may include primary care consultations, prescriptions, secondary care contacts and over-the-counter purchases by participants. Information on health care resource use and work absenteeism will be collected from participant questionnaires at six months. The robustness of the results will be explored using sensitivity analysis. Uncertainty in the confidence to be placed on the results of the economic analysis will be explored conducting a probabilistic sensitivity analysis to estimate cost effectiveness acceptability curves. Results from a broader societal perspective will also be presented, and will include participant-incurred and productivity costs in addition to heath care costs.

#### Qualitative study

A sample of responders to the 2-month follow up questionnaire are invited for interview to ask them about their experiences of participating in the trial and of living with hip osteoarthritis. Participants are sampled on a range of characteristics including age, gender, pain score and satisfaction with treatment. Participants are asked to complete and return a reply slip should they be interested in taking part in the interview. Interested participants are contacted by telephone to arrange a convenient time for the interview. Informed consent is taken prior to interview. Interviews are conducted either over the telephone or face-to-face depending on the participant’s preference. Recruitment is ongoing throughout the trial in phased batches.

#### Qualitative analysis

Data will be stored, and analysis managed, using NVivo software. All interviews are audio-recorded and fully transcribed verbatim and anonymised. An inductive approach will be taken to the analysis. Analysis is on-going from the first data collection time-point; the data is analysed thematically and a coding framework developed incorporating emergent themes. The data will undergo repeated comparisons through coding, recoding and memo writing in order to generate themes and concepts [28, 29], drawing on recognised techniques including the scrutiny of deviant cases, and checking for confirmatory or challenging evidence within the dataset [30]. Initial qualitative data analysis will be undertaken blind to the clinical trial results to facilitate an interpretive and inductive approach [31].

#### Patient and public involvement and engagement (PPIE)

This study was discussed with our large and active PPIE group prior to the funding submission. The group identified the importance of developing the research base for treatments that may provide an alternative to oral analgesia and that are less invasive than surgery. The group informed the design of the best current treatment intervention, as an active treatment arm involving advice on exercise and weight loss. The group have also piloted the time taken to complete baseline data collection and advised on content of the PIL and best current treatment information leaflets. The PPIE group will continue to work with the research team throughout the trial (for example in advising the dissemination plan).

#### Trial organisation and monitoring

The Trial Steering Committee met prior to ethics application in order to agree the final protocol, and at agreed time intervals over the course of the pilot trial. An independent Data Monitoring Committee (DMC) approved the protocol and reviews the safety of the trial. Detailed reports focusing on interim safety, recruitment and retention are prepared by Keele CTU at approximately 6 monthly intervals. All data collection, database design, data input and cleaning, as well as trial oversight procedures, are in line with the standard operating procedures of the Keele CTU and the conditions of the grant. Data is centrally monitored for quality and completeness by Keele CTU.

#### Data confidentiality and archiving

All information collected during the course of the trial is kept strictly confidential. Information is held securely on paper and managed electronically by Keele University through Keele CTU. Keele CTU comply with all aspects of the 1998 Data Protection Act. If a participant withdraws consent from trial intervention and/or further collection of data, their data will remain on file and is included in the final study analysis. At the end of the trial, data will be securely archived in line with the Sponsor’s procedures for a minimum of 5 years. Data held by Keele CTU will be archived in the designated Keele CTU archive facility and site data and documents will be archived at the participating sites. Following authorisation from the Sponsor, arrangements for confidential destruction will then be made.

#### Progress of the trial

Recruitment commenced in January 2016. Unfortunately, review of recruitment 3–6 months into the trial revealed that recruitment was sub-optimal and less than 50% of that predicted. Two key amendments were made (detailed in Table 1).

First on 19th August 2016 the eligibility criteria were amended. Criterion 3 was originally ‘Moderate-to-severe hip pain (a score of four or more on a 0-10 numeric rating scale (NRS)) on the day of assessment’. During the first 5 months of recruitment we observed that, due to the day-to day variability of osteoarthritis symptoms, a number of potential participants did not meet the eligibility criteria of pain of 4/10 on the day of assessment. The TSC suggested amending the criterion to ‘moderate to severe hip pain (a score of four or more on a 0-10 numeric rating scale (NRS)) on average over the last two weeks and current hip pain rated as at least one out of 10 (on a 0-10 NRS) on the day of assessment’. This amendment was approved by the DMC and Research Ethics Committee (REC). Forty-eight participants had been recruited at the time of this amendment.

Second, in March 2017, the Data Monitoring Committee noted poor recruitment and suggested rerunning the sample size calculations to ensure the original sample size assumptions were still valid. The observed baseline standard deviation (SD) of the primary outcome based on data collected from participants recruited by this time point (*n* = 65) was 1.7 (and the SD for follow up scores is around 2.5) – i.e. much lower than the SD of 4.5 expected before the start of recruitment on which the original sample size calculation was based. The clinically important difference of 1.5 (originally stated) in the context of this baseline SD would be ‘large’ (effect size above 0.8). The clinically important difference of 1.5 was considered to be too large in relation to the lower expected SD. The clinically important difference for the NRS-pain scale has taken different values across studies; an absolute difference of 1 has been specified in some studies (which would relate to a “moderate” effect size (0.5) when the SD is around 2; or, 0.4 in relation to higher SD of 2.5 which is observed across follow up time points). Hence, we felt that a revised effect size of 0.4 is justifiable. Using this revised effect size of 0.4 and revised power of 80% (on the advice of the TSC), the sample size was amended as described under ‘sample size’ heading, and was approved by the funder, TMC and DMC. A funded extension was requested, to extend recruitment from 18 months to 29 months, and approved in November 2017.

We removed a second qualitative objective to explore reasons for non-participation in the trial, to address any modifiable barriers to recruitment. This was in part due to limited capacity within the research team and low recruitment to this qualitative study of people who were eligible for the trial but unwilling to participate. All amendments have received ethical approval.

Recruitment via Route 3 commenced 12 months into recruitment and was noted to be more successful and became the principle route of recruitment from July 2017.

## Discussion

This paper describes the design of pragmatic randomised trial which investigates the comparative clinical and cost effectiveness of corticosteroid injections in reducing pain in people with hip OA. A number of issues have been addressed in the design of this trial.

As the majority of patients with hip OA are managed exclusively in primary care, it is important that the evidence base for recommended interventions is suitable for patients treated in this setting, especially at a time when clinical commissioning groups in the UK are redesigning NHS clinical pathways. The study participants in the existing five RCTs which have evaluated the effectiveness of corticosteroid intra-articular injections recruited participants from secondary care and are unlikely to be representative of the wider primary care population. Furthermore, three performed injections under fluoroscopic guidance which is not available in primary care [13, 14, 16].

Eligibility criteria were defined to recruit a representative primary care population of moderate to severe hip OA patients, protect patient safety and also to ensure maximum generalisability of the results to primary care. We deliberately did not exclude patients who were considered by clinicians to be eligible for onward referral for surgery. This decision was taken on the advice of our TSC, and particularly two lay members of the committee, who felt it was important that patients with more severe OA should still be offered choice of non-surgical interventions.

Whilst conducting the trial in a setting that is very close to primary care is crucial to our research question and to optimizing the generalisability of the findings, we recognised the need to maximise recruitment and achieve realistic recruitment targets. Recruitment to this trial within the first 5 months was lower than half that expected and amendments to the study eligibility criteria and recruitment routes are addressing this.

In summary, this paper describes the rationale and design for a randomised pragmatic trial that aims to determine the clinical and cost effectiveness of ultrasound guided intra-articular corticosteroid injections in hip OA. The proposed trial will make an important contribution to the evidence base available to support effective conservative management of hip OA in primary care and will inform both patient management and future research for treatment options for hip OA.

## Abbreviations

AP: Anteroposterior
ASIS: Anterior Superior Iliac Spines
BMI: Body Mass Index
CI: Chief Investigator
CTU: Clinical Trials Unit
DMC: Data Monitoring Committee
EULAR: European League Against Rheumatism
GAD: Generalised Anxiety Disorder
GCP: Good Clinical Practice
GP: General Practitioner
HIT: Hip Injection Trial
ID: Identification
ITT: Intention To Treat
MAR: Missing at random
MDC: Minimum data collection
MHRA: Medicines and Healthcare Products Regulatory Agency
MI: Multiple imputation
MMRM: Mixed models for repeated measures
MNAR: Missing not at random
MSP: Median sagittal plane
NHS: National Health Service
NICE: National Institute for Health and Care Excellence
NNT: Number needed to treat
NRS: Numeric Rating Scale
NSAID: Non-steroidal anti-inflammatory drugs
OA: Osteoarthritis
PI: Principal Investigator
PIL: Participant Information Leaflet
PPIE: Patient and Public Involvement and Engagement
QALY: Quality-adjusted life years
QoL: Quality of Life
RCT: Randomised Controlled Trial
REC: Research Ethics Committee
RfPB: Research for Patient Benefit
SAE: Serious Adverse Event
SD: Standard Deviation
SOP: Standard Operating Procedure
SPC: Summary of Product Characteristics
SUSAR: Suspected Unexpected Serious Adverse Reaction
THR: Total Hip Replacement
TSC: Trial Steering Committee
UK: United Kingdom
US: Ultrasound
WOMAC: Western Ontario and McMaster University Arthritis Index
